# Effects of Corticosteroid Treatment on Mycophenolic Acid Exposure in Renal Transplant Patients—Results From the SAILOR Study

**DOI:** 10.3389/fphar.2021.742444

**Published:** 2021-09-14

**Authors:** Nima Nourbakhsh, Jana Ekberg, Karin Skov, Christian Daugaard Peters, Aygen Øzbay, Per Lindner, Niels Henrik Buus

**Affiliations:** ^1^ Department of Renal Medicine, Aarhus University Hospital, Aarhus, Denmark; ^2^ Department of Transplantation, Sahlgrenska Hospital, Gothenburg, Sweden

**Keywords:** mycophenolic acid, mycophelonate mofetil, corticosteroids, renal transplantation, interaction, tacrolimus, prednisolone

## Abstract

**Background:** Mycophenolic acid (MPA) is a potent immunosuppressive agent used in solid organ transplantation. MPA exhibits large interindividual variation in dose-normalized plasma concentrations but is nevertheless usually prescribed as a fixed dose without use of therapeutic drug monitoring (TDM). Data on the effect of corticosteroid (CS) treatment on MPA concentrations during concomitant tacrolimus treatment remains sparse.

**Methods:** Data is based on TDM of MPA area under the concentration curve (AUC) in 210 renal transplant recipients participating in the prospective, randomized, controlled, multi-center trial (SAILOR) where a steroid-free immunosuppressive regimen with mycophenolate mofetil (MMF) and low-dose tacrolimus was compared with a conventional prednisolone-based treatment regimen. Multilevel mixed-effects linear regression post-hoc analyses of MPA AUC was performed.

**Results:** Median MPA AUC at baseline (within the first 2 weeks post-transplant) in patients taking 2 g MMF daily was 53 mg*h/L (interquartile range: 43–69 mg*h/L, min: 24—max: 117 mg*h/L). Between-patient variation in MPA AUC was up to 5-fold on the same MMF dose. Patients in the steroid-free group had 12.5% lower (95% CI; 3.2–20.9%, *p* = 0.01) MPA AUC levels at baseline compared to the steroid treated group. During follow-up (14 days–2 years post-transplant) there were no significant differences in MPA AUC between the groups with MPA AUC being 4.2% lower (95% CI: −4.8%−12,5%, *p =* 0.35) in the steroid-free *vs* standard treatment group in restricted analysis after multivariate adjustment for tacrolimus trough level, body weight, time after transplantation and MMF dose. MMF dose was positively correlated with MPA AUC (*p* < 0.001) whereas body weight was negatively correlated with MPA AUC (*p* < 0.001). MPA AUC was 0.4% (95% CI: 0.2–0.6%, *p* < 0.001) lower per 1 kg increase in weight. Tacrolimus trough levels had no significant effect on MPA AUC.

**Conclusion:** Immunosuppression with CS during concomitant tacrolimus treatment was shortly after transplantation associated with a significantly higher MPA exposure but the effect was small and not maintained during follow-up. Low body weight was associated with higher MPA exposure, which suggests a potential for weight adjusted MMF dosing.

## Introduction

Mycophenolate mofetil (MMF) has been an integral part of immunosuppressive treatment in organ transplantation for the past 20 years (Lim, Kohli, and Bloom 2017). MMF is a pro-drug hydrolyzed to its active form mycophenolic acid (MPA) rapidly after oral administration (Kuypers et al., 2010). It inhibits inosine monophosphate dehydrogenase, the rate limiting enzyme in the *de novo* synthesis of guanosine nucleotides, important for T- and B-lymphocyte function (Allison and Eugui 2000). MPA is mainly metabolized by uridine 5′-diphospho (UDP) -glucuronosyltransferases in the liver, intestine and kidney to the inactive metabolite MPA glucuronide (MPAG). MPA can be measured in plasma and a concentration peak is seen 1–2 h after oral administration of MMF with a possible second peak due to enterohepatic recirculation of both MPA and MPAG (Shaw et al., 2003; Bullingham, Nicholls, and Kamm 1998; Rong et al., 2019; Kuypers et al., 2003). The best assessment of systemic MPA exposure is the area under the plasma concentration curve (AUC), which like trough levels also demonstrates wide variability with more than ten-fold difference between-subjects for the same dose (Kuypers et al., 2010).

MPA exposure may be affected by co-administration of other immunosuppressive agents, such as cyclosporine, which seems to decrease MPA AUC (Gaston et al., 2009; Pou et al., 2001; Benjanuwattra, Pruksakorn, and Koonrungsesomboon 2020). On the contrary tacrolimus has less effect on MMF pharmacokinetics (Kagaya et al., 2008; Van Gelder et al., 2001; Kim et al., 2018; Nashan et al., 2009). Data regarding the influence of corticosteroids (CS) on MMF pharmacokinetics are limited and not uniform. CS could possibly increase hepatic clearance of MPA by induction of UDP-GT enzyme activity responsible for conversion of MPA to its inactive metabolite (Cattaneo et al., 2002; Staatz and Tett 2007; Rong et al., 2019; Benjanuwattra, Pruksakorn, and Koonrungsesomboon 2020). A prospective study on MPA exposure in combination with tacrolimus and CS found no change in MPA AUC after CS withdrawal (Kuypers et al., 2003). Another prospective study found that CS tapering and withdrawal in an MMF and cyclosporine based regimen led to increased MPA AUC and trough levels (Cattaneo et al., 2002). A more recent study based on a pharmacokinetic model suggests a reduction of MMF dosage in steroid-free regimens (Rong et al., 2019). A possible effect of CS on MPA pharmacokinetics during co-therapy with tacrolimus has not been investigated in large scale studies. This topic is important as triple therapy with tacrolimus, MMF and CS or dual therapy with tacrolimus and MMF are the two main immunosuppressive therapy regimens applied in kidney transplantation.

The present investigation is based on the Trial of Steroid Avoidance and Low-dose CNI by ATG-induction in Renal Transplantation (SAILOR). This randomized, clinical trial compared the effects of steroid-free immunosuppression (MMF and prednisolone) versus standard triple therapy (MMF, tacrolimus and prednisolone) on new onset diabetes following renal transplantation (Ekberg et al., 2014). Using data from the SAILOR cohort, the aim of the present substudy was to examine the effect of CS on MPA exposure during co-therapy with tacrolimus within the first 2 weeks post transplantation and the following 2 years.

## Methods

### Patients

SAILOR was a prospective, randomized, multi-center, controlled, open-label study involving kidney transplant recipients with a 2-year follow-up. SAILOR assessed the cumulative incidence of post-transplantation diabetes by comparing a steroid-free immunosuppressive protocol with a conventional immunosuppressive protocol (Ekberg et al., 2014). Patients older than 18 years at low immunological risk, receiving their first or second single-organ AB0-compatible kidney transplant from living or deceased donor were included. Recipients with more than 25% panel reactive HLA antibodies or those considered to be high risk of rejection or likely to need corticosteroid were excluded. A total of 222 patients (165 males, 57 females) were enrolled in the study from three transplant centers in Sweden (Gothenburg: *n* = 131; Malmö: *n* = 17) and Denmark (Aarhus: n = 74). The primary results from SAILOR will be presented in a separate publication, while the present substudy focuses on the effects of CS treatment on MPA exposure in terms of MPA AUC measurements.

The participants were randomized into two arms. Patients in the steroid-free arm (*n* = 113) received treatment with anti-thymocyte globulin induction (ATG (Thymoglobuline®, Sanofi AB)) 2.5 mg/kg on day 0 and 1 with 250 mg (day 0) and 50 mg methylprednisolone (day 1) bolus (Solu-Medrol®, Pfizer) before each ATG-dose. Maintenance therapy consisted of prolonged released tacrolimus (Advagraf®, Astellas Pharma) with a target of 5–10 ng/ml within the first 3 months and 4–7 ng/ml afterwards, and MMF with a starting dose of 1 g twice daily and later adjusted according to a target AUC of 40–60 mg*h/L. Standard arm patients (*n* = 109) were treated with basiliximab induction (Simulect®, Novartis) 20 mg on day 0 and 4, and 250 or 500 mg methylprednisolone (Solu-Medrol®, Pfizer) before reperfusion according to local practice. Maintenance treatment consisted of tacrolimus and MMF as in the steroid-free arm with prednisolone tapering according to practice of the local transplantation center, but not less than 5 mg daily.

The prednisolone treatment regimen in the two Swedish centers began with an initial dose of 100 mg followed by daily dose reduction of 20 mg until a daily dose of 20 mg. The subsequent tapering until 5 mg daily was carried out over the course of the following months. The regimen for the Danish patients started with a prednisolone dose of 20 mg daily tapered to 5 mg over the subsequent months. Patients with histology-proven rejection were treated with corticosteroids regardless of study arm and in accordance with local instructions.

### Blood Sampling and Pharmacokinetics

Among the 222 patients in SAILOR, 210 patients had one or more MPA AUC measurements. Most participants had a measurement within the baseline period (<14 days after transplantation). MPA AUC determinations during follow-up (14 days–2 years post-transplant) were made according to decisions from the treating physicians. The typical reason for a measurement was a change in MMF dose to ensure the patient was still within (or close to) the therapeutic range.

MPA AUC were determined using a limited sample strategy. Venous blood samples used for calculation of MPA-AUC_0-12_ were taken at time 0 (pre-dose), 30 min and 2 h after intake of the capsules. MPA-AUC_0-12_ were calculated from these three blood samples using the following validated model (Pawinski et al., 2002; Kuypers et al., 2003):

AUC = 7.75 + (6.49*C0h)+ (0.76*C0.5h)+ (2.43*C2h)

MPA concentrations were determined using an enzyme immunoassay method (CEDIA® Mycophenolic acid-analysis). The lower limit of detection of the immunoassay is 0.2 μg/ml. The first MPA AUC measurement was made within 2 weeks after transplantation (baseline). Subsequent measurements were done according to local practice or when prompted by investigators as explained above. MMF doses were recorded simultaneously with MPA AUC measurements. Tacrolimus trough levels were only recorded when it resulted in a tacrolimus dose change. Glomerular filtration rate (mGFR) was measured at 12 and 24 months using ^51^chrome-EDTA- or iohexol plasma clearance (Ekberg et al., 2014).

### Statistical Analysis

All statistical analyses were performed using Stata (Stata/IC version 16.1, Copyright 1985–2019 StataCorp LC, Texas 77,845 United States). *p*-values < 0.05 were considered statistically significant. Categorical patient characteristics data were compared using the Pearson chi-squared test and continuous data were analyzed using the independent *t*-test. Wilcoxon rank-sum test was used for non-normally distributed data. The assumption of normality was checked with QQ-plots and analyses were performed using natural log-transformation due to skewness.

For the main analysis multilevel mixed-effects linear regression was used for examining the effect of CS on MPA AUC. Adjustments were made for MMF dose, blood tacrolimus trough levels, time after transplantation and body weight.

## Results

### Patients

Patient characteristics were comparable in the two treatment arms except for a higher number of smokers and previous smokers in the steroid-free arm. There were no significant differences between the groups regarding mGFR at 12 and 24 months of follow-up (Table 1). Plasma albumin concentrations were only available in the 74 patients from Denmark. There were no difference in p-albumin levels between patients in the steroid-free arm and the standard treatment arm (Table 1).

**TABLE 1:** Patient characteristics and demographics.

Variable	Steroid-free arm (*n* = 113)	Standard arm (*n* = 109)	*p*-value
Age, years	52.1 (13.9)	49.2 (14.5)	0.1
Living donor	50 (44.3%)	41 (37.6%)	0.3
Male	83 (73.5%)	79 (72.5%)	0.9
Weight, kg	79.4 (15.6)	81.9 (17.2)	0.3
Height, cm	174.9 (9.8)	176.3 (9.9)	0.3
BMI, kg/m^2^	25.9 (3.9)	26.2 (4.0)	0.5
Active smokers	13 (11.6%)	4 (3.7%)	0.03
Ever smoked	48 (42.9%)	32 (29.3%)	0.04
mGFR 12 months, ml/min/1.73 m^2^	53.6 (17.0)	55.0 (16.6)	0.6
mGFR 24 months, ml/min/1.73 m^2^	52.5 (18.0)	54.5 (17.8)	0.4
*P-albumin at baseline, g/l	33.1 (4.3)	34.5 (4.3)	0.17

Data in n (% of total) or mean (standard deviation). BMI: body mass index. mGFR: measured glomerular filtration rate; *P-albumin: plasma-albumin (only available in the 74 patients from Denmark).

**FIGURE 1:**
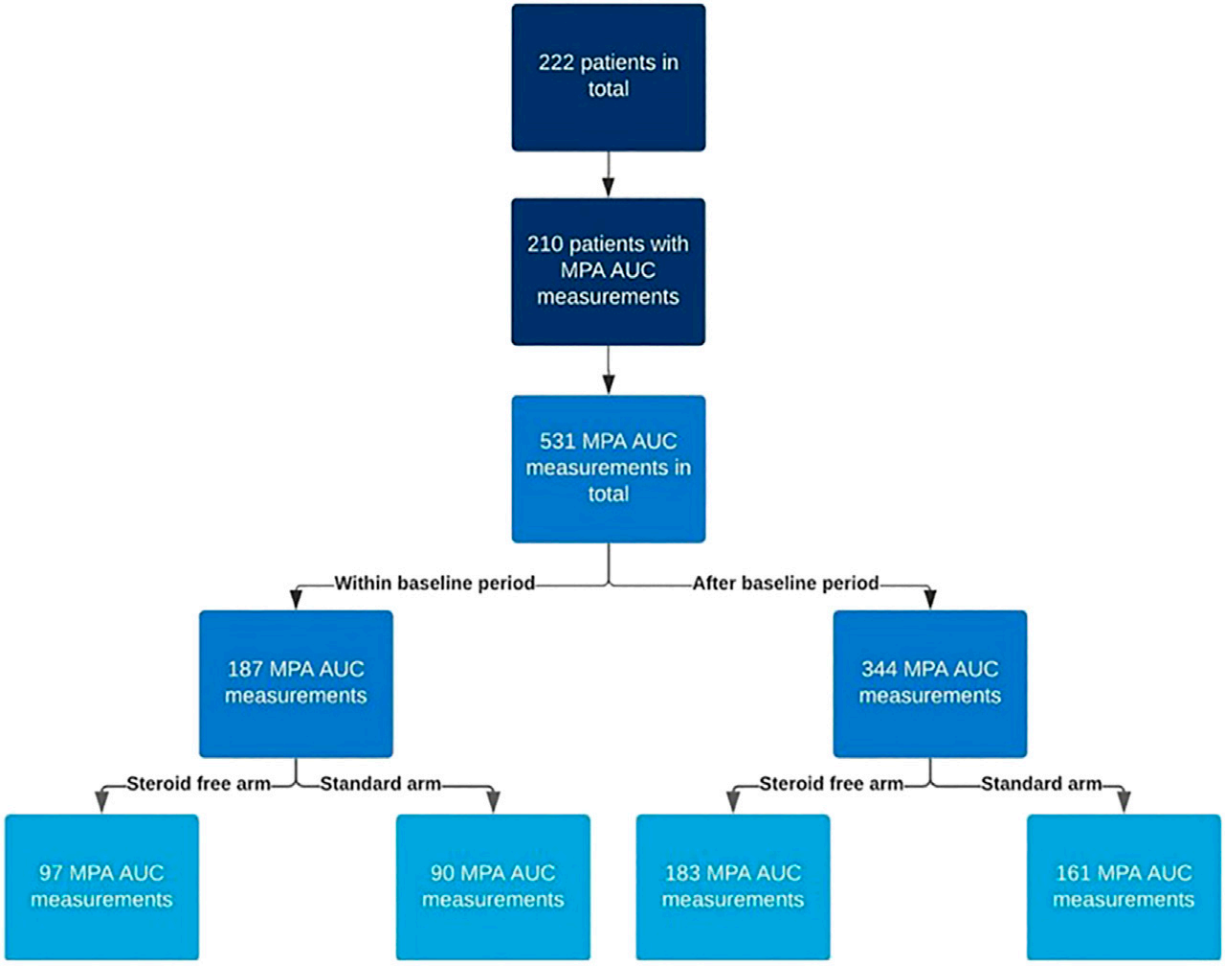
Flow chart describing the number of mycophenolic acid area under the curve (MPA AUC) measurements in the two treatment arms (steroid-free and standard). The baseline period is the first 2 weeks after transplantation.

**FIGURE 2:**
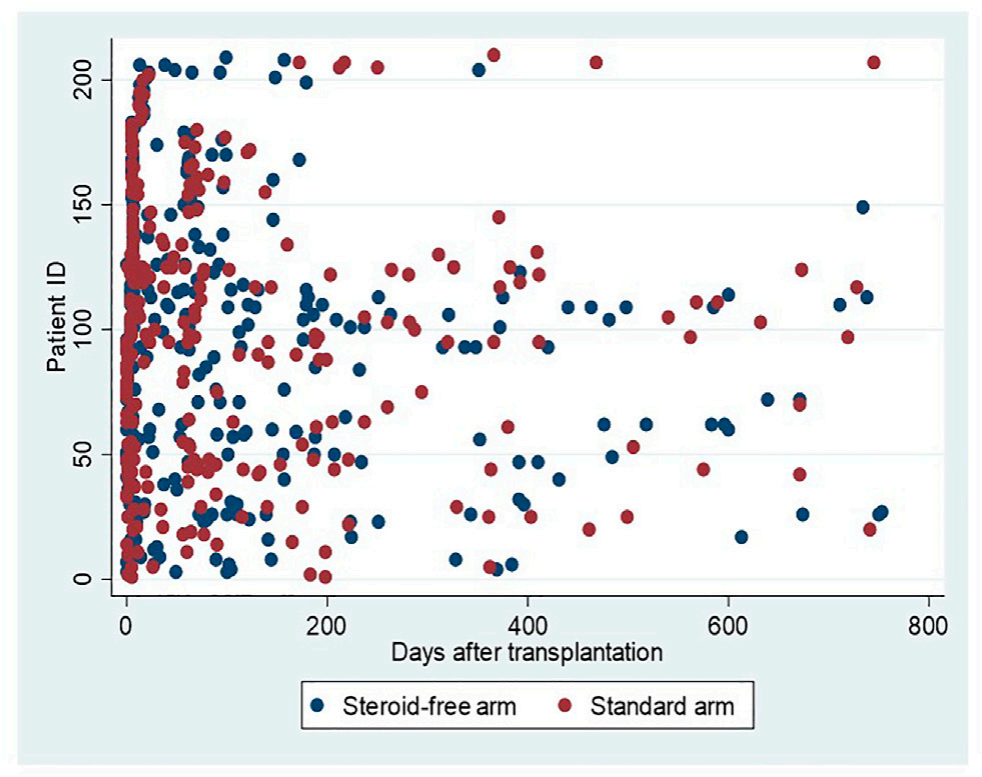
MPA AUC measurements in relation to time after transplantation. Blue dots represent measurements in patients allocated to the steroid-free arm and red dots measurements in patients allocated to the standard arm.

The flow chart in Figure 1 depicts the number of MPA AUC measurements in the two treatment arms. Figure 2 illustrates the number and time points of MPA AUC measurements for each patient during the 2-year study period. The number of MPA AUC measurements varied between 1 and 9 with an average of 2.5 measurements per patient. A total of 531 MPA AUC measurements were available, of which 280 were from the steroid-free group. However, 69 of these 280 measurements (24.6%) were performed during simultaneous steroid treatment. Temporary CS treatment in the steroid-free arm was primarily due to rejection (48 MPA measurements), MMF-intolerance (13 MPA measurements) or other reasons such as gout (8 MPA measurements). Patients in the steroid-free arm temporary receiving CS did not differ from the rest of the steroid-free group regarding age, sex, weight, or BMI.

### MPA AUC at Baseline

**FIGURE 3:**
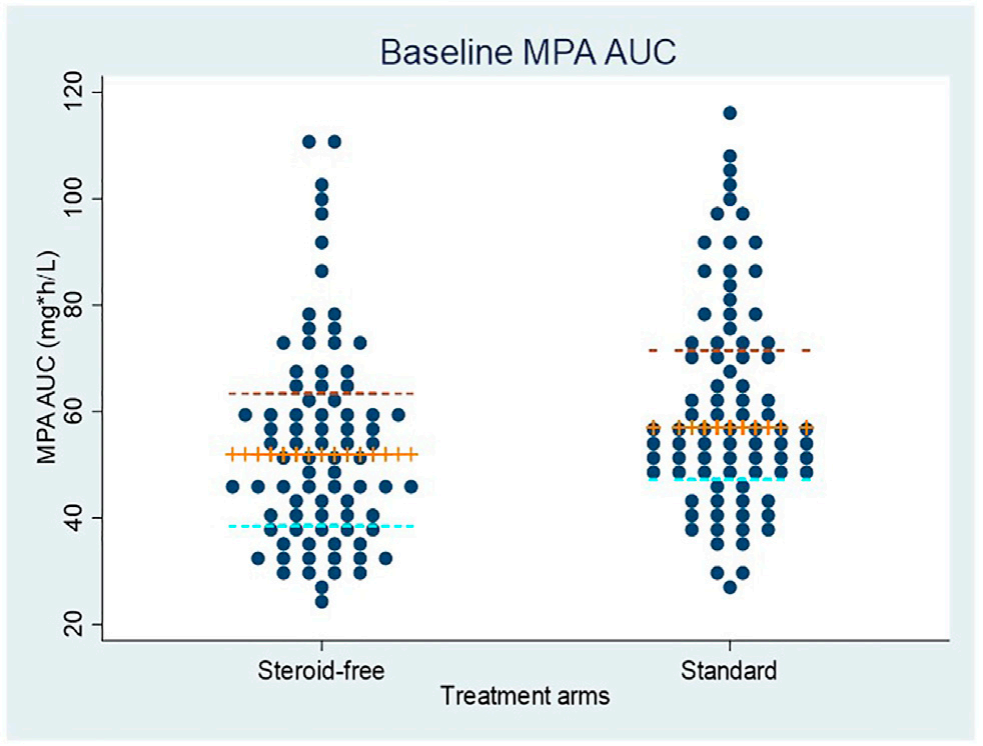
MPA AUC measurements at baseline (first 2 weeks after transplantation) in patients allocated to the steroid-free treatment arm and the standard treatment arm. All patients received a dose of mycophenolate mofetil of 1 g twice daily. The median value and interquartile ranges are shown by the horizontal lines.

MPA AUC measurements for each patient at baseline are shown in Figure 3 for the two treatments arms. Baseline is defined as the first 2 weeks post-transplantation where all patients received an MMF dose of 1 g twice daily. Among the 210 patients with MPA AUC measurements there were 187 measurements within the baseline period. The median MPA AUC at baseline for these 187 measurements was 53 mg*h/L (IQR: 43–69 mg*h/L, min: 24—max: 117 mg*h/L). Despite a high interindividual variation of the MPA AUC values, a small but significant difference was found between the groups, with a lower median AUC of 52 mg*h/L (IQR: 39–64 mg*h/L, min: 24—max: 110 mg*h/L) in the steroid-free group as compared to 57 mg*h/L (IQR 48–72 mg*h/L, min: 28—max: 117 mg*h/L) in the standard treatment group (Figure 3). Back-transformation of natural logarithmic data showed that MPA AUC at baseline was 12.5% lower (95% CI; 3.2–20.9%, *p* = 0.01) in the steroid-free arm compared to the standard treatment arm.

**FIGURE 4:**
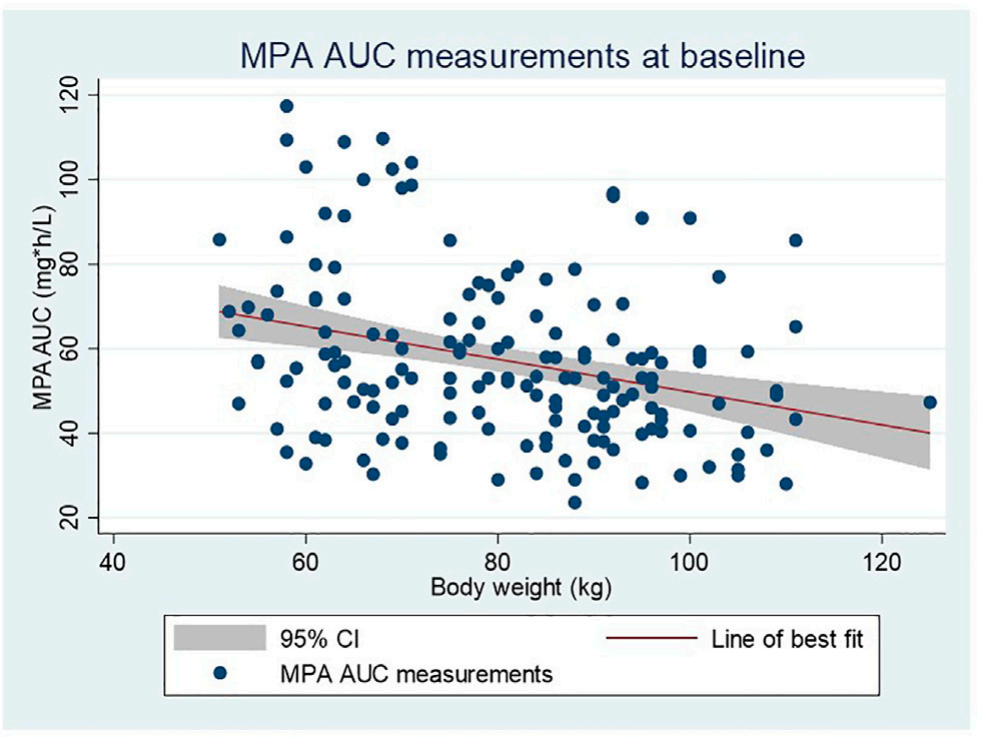
Linear regression showing the relationship between MPA AUC measurements and body weight in all patients at baseline (first 2 weeks after transplantation). All patients received a dose of mycophenolate mofetil of 1 g twice daily.

The association between MPA AUC measurements and body weight at baseline is shown in Figure 4. The linear regression coefficient is −0.32 (95% CI: −0.50; −0.14, *r* ^2^ = 0.07) with *p* = 0.001. A similar significant association was found when using body surface area (BSA) instead of weight.

### Corticosteroid Effect on MPA AUC

Table 2 shows results from the multilevel mixed-effects linear regression analysis based on all available MPA AUC measurements in the study regardless of timepoint and concomitant steroid treatment. Although not statistically significant patients in the steroid free group (*n* = 113) tended to have lower MPA AUC levels (*p =* 0.16) when adjusted for blood tacrolimus trough level, body weight, time after transplantation and MMF dose compared to the steroid treated group. Back-transformation of natural logarithmic data showed that MPA AUC levels in the steroid-free arm were 4.9% lower (95% CI: −2.0%–11.4%, *p* = 0.16) compared to the standard treatment arm after adjustment for the factors mentioned above.

**TABLE 2:** Multilevel mixed-effects linear regression based on all available MPA AUC measurements (*n* = 531).

CS effect on MPA AUC—all measurements			
lnAUC	β ln (mg*h/L)	[95% conf. Interval]	*p*-value
Steroid-free arm	−0.051	−0.12 to 0.02	0.16
MMF (mg)	0.00030	0.00024 to 0.00036	<0.001
TAC trough level (ng/ml)	−0.0038	−0.011 to 0.0038	0.33
Weight (kg)	−0.0043	−0.0064 to −0.0021	<0.001
Time after transplantation (months)	0.0065	0.0017 to 0.011	0.01

lnAUC = natural log transformation of AUC.

When omitting samples taken during concomitant steroid treatment in the steroid free arm the difference in MPA AUC between the two treatment arms increased and became statistically significant so that allocation to the steroid-free group was associated with significantly lower MPA AUC level compared to the steroid-treated group when adjusted for blood tacrolimus trough level, body weight, time after transplantation and MMF dose (*p* = 0.04) (Table 3). Back-transformation showed that MPA AUC levels in the steroid-free arm were 7.4% lower (95% CI: 0.5%–13.9%, *p* = 0.04) compared to the standard steroid treated arm in the adjusted analysis omitting samples taken during concomitant steroid treatment in the steroid free arm.

**TABLE 3:** Multilevel mixed-effects linear regression using natural log transformation based on all MPA measurements except those in the steroid-free arm performed on concomitant steroid therapy (*n* = 462).

CS effect on MPA AUC - no concomitant steroid in the steroid-free arm			
lnAUC	β ln (mg*h/L)	[95% conf. Interval]	*p*-value
Steroid-free arm	−0.077	−0.15 to −0.0045	0.04
MMF (mg)	0.00028	0.00021 to 0.00035	<0.001
TAC trough level (ng/ml)	−0.0043	−0.012 to 0.0036	0.28
Weight (kg)	−0.0049	−0.0071 to −0.0027	<0.001
Time after transplantation (months)	0.0072	0.0017 to 0.013	0.01

lnAUC = natural log transformation of AUC.

When restricting the analysis to the 274 MPA AUC measurements made after the baseline period and still omitting MPA AUC measurements in the steroid-free arm measured during concomitant steroid treatment, the difference became insignificant. Back-transformation showed that patients in the steroid-free treatment arm had MPA AUC 4.2% lower (95% CI: −4.8%−12,5%, *p =* 0.35) compared to the standard treatment arm.

We found no significant effect of tacrolimus trough levels on MPA AUC, neither in the analysis containing all MPA AUC measurements regardless of concomitant steroid treatment in the steroid free group (Table 2) nor in the analysis omitting these measurements (Table 3). Increasing MMF dosage was significantly associated to higher AUC regardless of using all measurements (*p* < 0.001) or omitting measurements with concomitant steroid use in the steroid-free arm (*p* < 0.001). Body weight was negatively correlated with MPA AUC level when using all available data (*p* < 0.001) and in the restricted analysis without concomitant steroid therapy (*p* < 0.001). Upon back-transformation MPA AUC when adjusted for MMF-dose, TAC trough level, time after transplantation and treatment allocation was 0.4% (95% CI: 0.2–0.6%, *p* < 0.001) lower per 1 kg increase in weight. Time after transplantation was positively correlated with higher AUC level and was also found to be a significant factor regardless of whether all samples were included or measurements with concomitant steroid use in the steroid-free arm were omitted (*p* < 0.01).

## Discussion

In this study data from the randomized controlled trial SAILOR were used to assess the influence of CS on MPA at baseline (2 weeks after transplantation) and during 2 years of follow-up. We found significantly increased MPA AUC levels in CS treated patients at baseline. However, based on the large interindividual variation of MPA AUC levels the net effect of CS is small, and no significant effects were detectable during follow-up. Furthermore, we found a consistent negative association between body weight and MPA AUC.

Our finding are in contrasts to previous studies by Cattaneo et al. (2002) and Rong et al. (2019) which both suggested a need for MMF dose reduction in patients on CS-free regimens. The pharmacokinetic model by Rong et al. based on data from 27 patients on steroid-free regimens showed significantly lower plasma MPA clearance compared to clearances reported from studies on CS treated patients. However, weight was not a significant covariate in the study by Rong et al*.* which they explain by a relatively small variability in this factor emphasizing the importance of sample size. Cattaneo et al. also found increased MPA AUC with concurrent tapering of methylprednisolone in 26 cyclosporine-treated patients despite unchanged MMF dosing (Cattaneo et al., 2002). MPA AUC was also measured at 21 months in a 12-patient control group receiving triple therapy with CS showing a significant difference between the steroid-free and the steroid-treated groups at 21 months. Cyclosporine decreases the enterohepatic recycling of MPA by inhibiting the biliary excretion of the MPA metabolite MPA-7-O-glucuronide leading to reduced reabsorption of MPA from the gastrointestinal tract causing lower MPA exposure (Staatz and Tett 2007; Benjanuwattra, Pruksakorn, and Koonrungsesomboon 2020). Concurrent cyclosporine treatment could have influenced the MPA concentrations with higher cyclosporine concentrations causing lower MPA concentrations. In the study cyclosporine AUC was lower in the control group at 21 months while MPA AUC in the control group was lower as well, which could indicate that cyclosporine was not causing the difference between the two groups. Consistent with previous reports tacrolimus trough levels in our study did not show any association with MPA AUC levels (Pou et al., 2001; Kagaya et al., 2008).

Although our results indicate that CS increase MPA exposure at baseline, no significant effects of CS were detectable during follow-up. This is partially in line with the results reported by Kuypers et al. (Kuypers et al., 2003) who found no significant effect of CS withdrawal on MPA AUC in 26 patients on a tacrolimus-based regimen.

Consistent with previous studies we observed large inter-individual variations in MPA AUC with a more than 5-fold difference between the lowest and highest values at baseline (Kuypers et al., 2010; Cattaneo et al., 2001; Johnson et al., 1999). This further substantiates the importance of a large sample size in examining MMF pharmacokinetics. Low patient weight was associated with higher MPA AUC measurements. This was a consistent finding in our study at baseline and in both multivariate models and is in agreement with findings from previous studies (Yau et al., 2007; Kaplan et al., 2010).

Although the variation in MPA exposure is known to be large, concentration-controlled dosing is rarely used and MMF is still most commonly administered as a predetermined standard dose (Gaston et al., 2009). According to recent reports from Metz et al*.* (Metz et al., 2019; Holford, Ma, and Metz 2020) this is largely due to the limitations of therapeutic drug monitoring (TDM). In TDM a range of concentrations is targeted, and the clinician is offered no dose recommendations. In the randomized controlled trials investigating fixed MMF dosing versus concentration-controlled dosing using TDM it was not possible to differentiate MPA exposure between the treatment and control arms. This resulted in similar MPA exposures and therefore also similar outcomes in both groups (Van Gelder et al., 2008; Gaston et al., 2009).

Studies using target concentration intervention (TCI) instead of TDM, where a specific concentration value is targeted rather than a range, with dose recommendations for the clinician through maximum a posteriori Bayesian estimations, have shown significantly reduced graft rejection among patients with TCI rather than fixed dosing, indicating the possible future use of concentration controlled dosing of MMF using TCI (Hale et al., 1998; Le Meur et al., 2007).

MMF dose is important for MPA exposure, with higher doses being associated with higher exposure even though this correlation is with substantial inter-individual difference as demonstrated by the 5-fold variation in exposure at baseline in our study. Significant factors contributing to this was weight and time after transplantation. However, also other factors may be important, and our study does not provide information on the influence of gastro-intestinal absorption, proton pump inhibitor use, ethnicity, or UGT-enzyme polymorphisms that could possibly have contributed to this variation. MPA exposure is also shown to be affected by plasma albumin concentrations with a risk of increased MPA exposure in patients with low albumin levels (Sheng et al., 2020). However, the limited number of plasma albumin measurements precluded us from including this factor in the regression analysis. Of notice, CS treatment did not seem to influence albumin levels in our study. Furthermore, albumin levels shortly after transplantation was only slightly reduced and will supposedly be close to normal during the follow-up period. MPA concentrations were measured with an immunoassay method, while the model used for calculation of MPA AUC by Pawinski et al. is determined by high-performance liquid chromatography measurements. This could possibly have introduced a measurement bias. However, because of randomization this error should be equal in both treatment arms. The renal clearance of MPA is only around 1% of total clearance and graft function is usually not considered of importance for MPA metabolism.

In conclusion, in our study based on 210 patients from the SAILOR cohort, CS use was associated with higher MPA AUC levels at baseline but not during follow-up. The SAILOR study was not originally designed to investigate this topic and certain limitations therefore apply to our investigation. These limitations were primarily in terms of variable numbers of MPA concentration measurements and that the measurements were performed at undefined time points. We recognize that a large proportion of MPA AUC measurements in the steroid-free group were performed during concomitant steroid treatment, which could possibly have masked a more substantial effect of CS on MPA exposure during follow-up. Nonetheless the magnitude of the CS effect is small and not likely to influence clinical decision regarding MMF dosage. The strengths of our study include a large number of patients and a randomized design with two different treatment arms, where one arm excluded daily use of steroids. The fact that weight was significantly associated with AUC suggests the potential for weight-adjusted dosing of MMF instead of a fixed dose. Important clinical implications of the present data set, achieved from daily practise, is an absence of a clinically significant interaction between CS treatment and MPA exposure, and a suggestion to consider body weight during MMF dosing. Further research is however required to fully clarify factors with possible influence on MPA exposure in order to guide individual MMF dose adjustments.

## Data Availability

The raw data supporting the conclusions of this article will be made available by the authors, without undue reservation.
